# Imaging the Meissner effect in pressurized bilayer nickelate with integrated multi-parameter quantum sensor

**DOI:** 10.1093/nsr/nwaf268

**Published:** 2025-07-04

**Authors:** Junyan Wen, Yue Xu, Gang Wang, Ze-Xu He, Yang Chen, Ningning Wang, Tenglong Lu, Xiaoli Ma, Feng Jin, Liucheng Chen, Miao Liu, Jing-Wei Fan, Xiaobing Liu, Xin-Yu Pan, Gang-Qin Liu, Jinguang Cheng, Xiaohui Yu

**Affiliations:** Beijing National Laboratory for Condensed Matter Physics and Institute of Physics, Chinese Academy of Sciences, Beijing 100190, China; School of Physical Sciences, University of Chinese Academy of Sciences, Beijing 100190, China; Beijing National Laboratory for Condensed Matter Physics and Institute of Physics, Chinese Academy of Sciences, Beijing 100190, China; School of Physical Sciences, University of Chinese Academy of Sciences, Beijing 100190, China; Beijing National Laboratory for Condensed Matter Physics and Institute of Physics, Chinese Academy of Sciences, Beijing 100190, China; School of Physical Sciences, University of Chinese Academy of Sciences, Beijing 100190, China; Beijing National Laboratory for Condensed Matter Physics and Institute of Physics, Chinese Academy of Sciences, Beijing 100190, China; School of Physical Sciences, University of Chinese Academy of Sciences, Beijing 100190, China; Beijing National Laboratory for Condensed Matter Physics and Institute of Physics, Chinese Academy of Sciences, Beijing 100190, China; Beijing National Laboratory for Condensed Matter Physics and Institute of Physics, Chinese Academy of Sciences, Beijing 100190, China; School of Physical Sciences, University of Chinese Academy of Sciences, Beijing 100190, China; Beijing National Laboratory for Condensed Matter Physics and Institute of Physics, Chinese Academy of Sciences, Beijing 100190, China; Beijing National Laboratory for Condensed Matter Physics and Institute of Physics, Chinese Academy of Sciences, Beijing 100190, China; School of Physical Sciences, University of Chinese Academy of Sciences, Beijing 100190, China; Beijing National Laboratory for Condensed Matter Physics and Institute of Physics, Chinese Academy of Sciences, Beijing 100190, China; School of Physical Sciences, University of Chinese Academy of Sciences, Beijing 100190, China; Beijing National Laboratory for Condensed Matter Physics and Institute of Physics, Chinese Academy of Sciences, Beijing 100190, China; Beijing National Laboratory for Condensed Matter Physics and Institute of Physics, Chinese Academy of Sciences, Beijing 100190, China; Department of Physics, Hefei University of Technology, Hefei 230009, China; Laboratory of High Pressure Physics and Material Science, School of Physics and Physical Engineering, Qufu Normal University, Qufu 273165, China; Beijing National Laboratory for Condensed Matter Physics and Institute of Physics, Chinese Academy of Sciences, Beijing 100190, China; School of Physical Sciences, University of Chinese Academy of Sciences, Beijing 100190, China; CAS Center of Excellence in Topological Quantum Computation, University of Chinese Academy of Sciences, Beijing 100190, China; Beijing National Laboratory for Condensed Matter Physics and Institute of Physics, Chinese Academy of Sciences, Beijing 100190, China; School of Physical Sciences, University of Chinese Academy of Sciences, Beijing 100190, China; CAS Center of Excellence in Topological Quantum Computation, University of Chinese Academy of Sciences, Beijing 100190, China; Beijing National Laboratory for Condensed Matter Physics and Institute of Physics, Chinese Academy of Sciences, Beijing 100190, China; School of Physical Sciences, University of Chinese Academy of Sciences, Beijing 100190, China; Beijing National Laboratory for Condensed Matter Physics and Institute of Physics, Chinese Academy of Sciences, Beijing 100190, China; School of Physical Sciences, University of Chinese Academy of Sciences, Beijing 100190, China

**Keywords:** quantum metrology, nickelates, high-temperature superconductivity, Meissner effect, high pressure

## Abstract

Recent reports on the signatures of high-temperature superconductivity with a critical temperature (*T*_c_) close to 80 K have triggered great research interest and extensive follow-up studies. Although zero resistance has been successfully achieved under improved hydrostatic pressure conditions, the Meissner effect of La_3_Ni_2_O_7–δ_ under high pressure remains controversial. Here, using shallow nitrogen-vacancy centers implanted on the culet of diamond anvils as *in situ* quantum sensors, we observe compelling evidence for the Meissner effect in polycrystalline bilayer nickelate samples: magnetic field expulsion during both field-cooling and field-warming processes. In particular, we explore the multi-parameter measurement capacity of the diamond quantum sensors to extract the weak demagnetization signal of La_3_Ni_2_O_7–δ_. The correlated measurements of Raman spectra and magnetic imaging indicate an incomplete structural transformation related to the displacement of oxygen ions emerging in the non-superconducting region. Our work clarifies the controversy about the Meissner effect of La_3_Ni_2_O_7–δ_ and contributes to the development of quantum sensing of weak signals under high-pressure conditions.

## Teaser

Diamond quantum sensing provides compelling evidence for the Meissner effect in nickelate high-temperature superconductors under high pressure.

## INTRODUCTION

Since superconductivity has been observed in Nd_1−x_Sr_x_NiO_2_ thin films, the study of nickelate superconductors has attracted substantial attention in the condensed matter physics community [1–5]. Notably, pressure plays a significant role in regulating the superconductivity of nickelates. It has been shown that the critical temperature (*T*_c_) of Pr_0.82_Sr_0.18_NiO_2_ thin films can be significantly increased to ∼31 K by applying hydrostatic pressure up to 12 GPa [6]. Recent studies have observed signatures of high-temperature superconductivity (HTSC) in pressurized single crystals of La_3_Ni_2_O_7–δ_, with an onset *T*_c_ of about 78 K, exceeding the McMillan limit and indicating a potential new class of HTSC at liquid nitrogen temperatures [7]. This significant discovery rapidly garnered widespread attention within the superconductivity research community [8–12]. With improved hydrostaticity, zero resistance has been researched [13–15], and angle-resolved photoemission spectroscopy (ARPES) [16,17] and optical conductivity [10] have been used to characterize its electronic properties. Meanwhile, resonant inelastic X-ray scattering (RIXS) [18], muon-spin relaxation (µSR) [19,20] and nuclear magnetic resonance (NMR) [21] have been used to reveal the pressure-driven transition of spin-density waves (SDWs) and charge-density waves (CDWs) in nickelates.

In contrast to the extensive and unambiguous evidence of its electronic properties, the final criterion of superconductivity, the Meissner effect, remains controversial for La_3_Ni_2_O_7–δ_ under pressure [7,22]. Using modulated ac susceptibility measurements, Zhou *et al*. found that the relative superconducting volume fraction in La_3_Ni_2_O_7_ is only 0.68%, suggesting that the superconductivity in this nickelate is filamentary-like [23]. Using superconducting quantum interference device (SQUID) magnetometry, Li *et al*. reported that the maximum superconducting volume fraction of La_3_Ni_2_O_7_ at 22.0 GPa reaches 62.7%, suggesting the bulk nature of superconductivity [24]. Such a large difference in superconducting volume fraction may result from a mixture of La_3_Ni_2_O_7_ and other phases such as La_4_Ni_3_O_10_ and La_2_NiO_4_. Indeed, using multi-slice electron ptychography and electron energy-loss spectroscopy, Li *et al*. found considerable inhomogeneity of oxygen content within the sample [25]. By replacing one-third of the La with Pr, Wang *et al*. demonstrated that the trilayer phase can be significantly suppressed, resulting in a superconducting volume fraction up to 97% in La_2_PrNi_2_O_7_ [26]. Accordingly, it is important to re-examine the Meissner effect of the bilayer nickelate La_3_Ni_2_O_7_.

In this work, we adapt the newly developed quantum sensing techniques with nitrogen-vacancy (NV) centers integrated into the diamond anvil cells (DACs) [27–32], assisted by correlated Raman spectra, to image the Meissner effect of bilayer nickelate under high pressure. With the spatially resolved magnetic field imaging around the nickelate samples, clear magnetic field expulsion effects are observed on a scale of tens of micrometers, indicating the bulk nature of superconductivity. In particular, to extract the weak demagnetization signal from the pressure-sensitive La_3_Ni_2_O_7_ sample, we explore the multi-parameter measurement capacity of the NV center and demonstrate the simultaneous imaging of pressure and magnetic field under high pressures. The correlated measurements of Raman spectra and NV-based magnetic imaging indicate an incomplete structural transformation related to the displacement of oxygen ions emerging in the non-superconducting region. Our results provide crucial evidence for the Meissner effect in pressurized bilayer nickelate superconductors and contribute both to the mechanistic understanding of their HTSC and to the development of NV-based quantum sensing under high-pressure conditions.

## RESULTS

**Figure 1.**
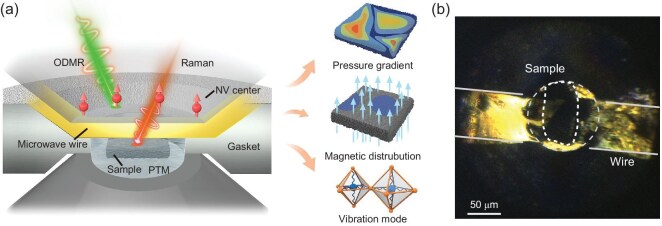
Correlated characterization of La_3_Ni_2_O_7–δ_ in DACs. (a) The La_3_Ni_2_O_7–δ_ and La_2_PrNi_2_O_7_ samples are wrapped in the pressure transmitting medium (PTM; silicone oil or KBr) inside the DAC sample chamber. Correlated measurements of NV-based quantum sensing and Raman spectra are performed to reveal the pressure gradient, magnetic distribution and vibration mode of the samples. This provides a direct method to study the Meissner effect of superconductors under pressure. For *in situ* magnetic field and pressure measurement, diamond anvils ([111]-crystal cut) with a layer of shallow NV centers are used, and a gold antenna is placed between the anvil culet and the PTM to transmit the microwave signal. (b) Bright-field image of the diamond culet after loading sample A with 20 GPa. The sample is outlined by a white dashed line.

As shown in Fig. 1a, the high-pressure sample chamber consists of two diamond anvils and a gasket. Through the transparent diamond window, optically detected magnetic resonance (ODMR) and Raman spectra can be measured under high pressure, in a correlated manner. To investigate the magnetic field and stress distribution around the superconducting samples, shallow NV centers are created on one of the diamond culets by nitrogen ion implantation (20 keV, 2 × 10^14^ cm^−2^) and subsequent high temperature annealing (800°C, 2 h). The spin resonance frequencies of an NV center are sensitive to the local magnetic field (and also to pressure, temperature and so on) and can be read out optically, providing a convenient and efficient method to study magnetic properties inside DACs. Details of the ODMR technique can be found in the Methods and previous work [29,33].

The Meissner effect of La_2_PrNi_2_O_7_ and La_3_Ni_2_O_7–δ_ polycrystalline samples was studied using diamond quantum sensors, in conjunction with *in situ* Raman measurements. For sample A, La_2_PrNi_2_O_7_ in liquid pressure-transmitting medium (silicon oil), ODMR spectra were measured under different pressures and external magnetic fields. We also performed experiments with La_2_PrNi_2_O_7_ (sample B) in a solid pressure-transmitting medium (KBr) to investigate the influence of hydrostatic pressure conditions on superconductivity. Correlated Raman and ODMR measurements were performed on sample B to determine the structural distinctions on both the superconducting and non-superconducting regions. Finally, sample C, La_3_Ni_2_O_7–δ_ in silicon oil, was measured. Since the diamagnetism signal of this La_3_Ni_2_O_7–δ_ sample is weak and buried in the pressure gradient background, we performed additional zero-field ODMR measurements to extract and decouple the contribution of local stress, and successfully obtained the diamagnetism image of the La_3_Ni_2_O_7–δ_ sample.

### Local diamagnetism in La_2_PrNi_2_O_7_

**Figure 2.**
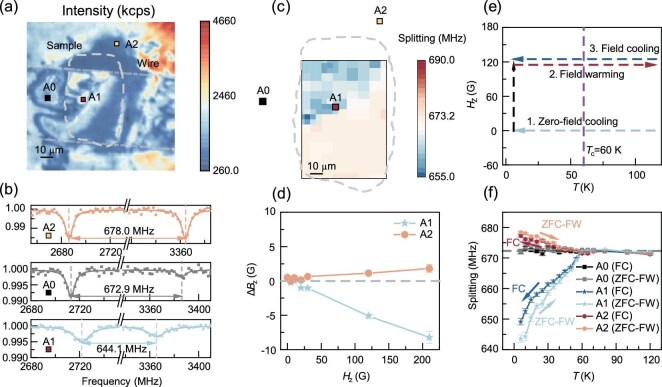
Local diamagnetism in La_2_PrNi_2_O_7_ at 20 GPa. (a) Fluorescence image of sample A (La_2_PrNi_2_O_7_ in silicon oil). The edge of the sample is outlined with a gray dashed line. The unit of the fluorescence intensity is kilo counts per second (kcps). (b, c) Typical ODMR spectra of the NV centers (b) and magnetic field mapping (c) under an external magnetic field of *H*_Z_ ∼ 120 G after ZFC of the sample to 7 K. Three points (A0, A1, A2) are selected based on their position with respect to the La_2_PrNi_2_O_7_ sample. Point A1 is directly on the sample, A2 is at the edge of the sample and A0 is far away from the sample (serves as a reference point). The blue area in (c) shows the local diamagnetism (these points have smaller ODMR splitting than that of the reference point). (d) Local magnetic field at the NV positions as a function of the applied external magnetic field (after ZFC). For the NV centers at point A1, their local magnetic field is about 5% smaller than the applied external magnetic field. In sharp contrast, the NV centers at point A2 feel an enhanced local magnetic field. (e) Measurement protocol of ZFC-FW and FC. (f) ODMR splitting of three selected points under 120 G ZFC-FW and FC measurements. The combination of the ZFC-FW and FC curves provide clear evidence of the Meissner effect.

We start with the sample of La_2_PrNi_2_O_7_ in silicon oil (sample A) at 20 GPa. As shown in Figs 1b and 2a, the sample edge and its relative position on the diamond culets were determined by comparing the bright-field image and the confocal NV fluorescence image; see Fig. S1 for more details. Figure 2b shows typical ODMR spectra of NV centers near the sample. These spectra were measured under an external magnetic field of around 120 G after zero-field cooling the sample to 6 K. The strength of the external magnetic field was calibrated using the ODMR splitting of the NV centers away from the sample (point A0 in Fig. 2a). For NV centers directly above the sample, e.g. at point A1, the ODMR splitting (644.1 MHz) is noticeably smaller than that of the reference point (672.9 MHz), indicating local diamagnetism of the La_2_PrNi_2_O_7_ sample. At the same time, a relatively larger splitting (678.0 MHz) was observed at point A2, which was due to the magnetic flux concentration at the sample edge. By measuring more points around the sample, a superconducting region can be identified, as shown by the blue color in Fig. 2c. For the measured region of 110 μm × 74.5 μm, about two-fifths of the points show diamagnetism, indicating a relatively large amount of superconducting shielding volume in the La_2_PrNi_2_O_7_ sample. In addition, Fig. 2d presents diamagnetism (at point A1) and flux concentration effects (at point A2) under different external magnetic fields, which show an almost linear dependence in all measured magnetic fields.

To further verify the Meissner effect of the La_2_PrNi_2_O_7_ sample, more ODMR spectra were acquired during zero-field-cooling field-warming (ZFC-FW) and field-cooling (FC) processes, with an external magnetic field of 120 G. The detailed experimental protocol is shown in Fig. 2e. As displayed in Fig. 2f, both the diamagnetism (at point A1) and the flux concentration (at point A2) show a clear temperature dependence. In comparison, the reference point (A0) remains almost constant splitting throughout the ZFC-FW and FC processes. From these results, a superconducting transition temperature of *T*_c_ ∼ 60 K is obtained and the expulsion of magnetic flux throughout the FC process, an intrinsic property of the Meissner effect, is observed. Meanwhile, compared to the ZFC-FW process, a weaker diamagnetism effect was observed at point A1 during the FC process, which can be attributed to the flux trapping effect [31].

To rule out the possibility that the diamagnetism originates from other magnetic impurities, both the ZFC-FW and FC measurements were performed under an external magnetic field of opposite direction and a strength of about −210 G (see Fig. 2e for the experimental protocol). The diamagnetism effect and its temperature dependence are similar to those observed at 120 G (shown in Fig. S2), indicating that the local diamagnetism sensed by the NV centers is not due to the compensating effect of local magnetic impurities in or near the samples. In addition, we varied the pressure from 11 to 30 GPa and performed ZFC-FW measurements. The reduced diamagnetic strength at pressures above 20 GPa indicates that superconductivity is gradually suppressed by the pressure (shown in Fig. S3). The *T*_c_ values determined from the ODMR measurements are included in the phase diagram in Fig. S3d. After the pressure decreased to 11 GPa, the effect of diamagnetism disappeared, which is consistent with the electrical resistance results in the previous study [26].

### Simultaneous magnetic and Raman measurements

**Figure 3.**
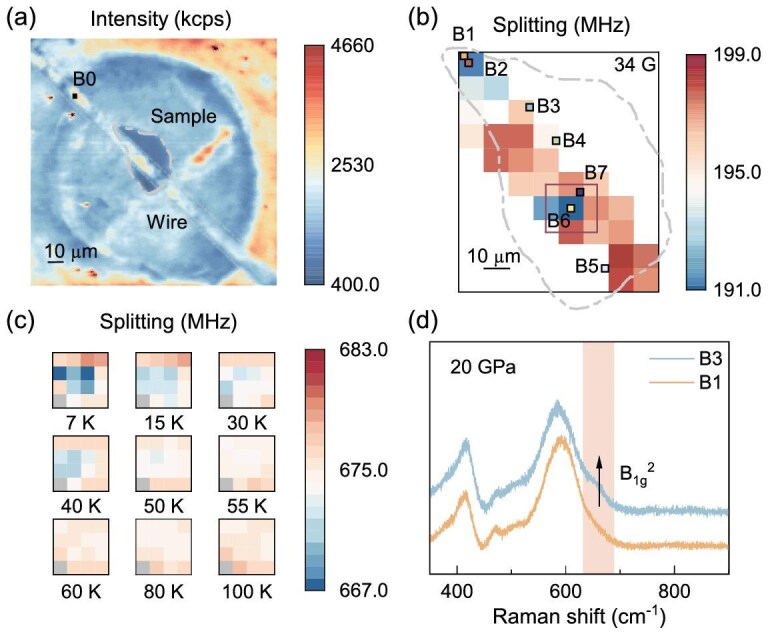
Correlated magnetic and Raman measurements of La_2_PrNi_2_O_7_ under pressure. (a) Fluorescence image of sample B (La_2_PrNi_2_O_7_ in KBr). The edge of the sample is outlined with a black dashed line. (b) Magnetic field mapping under an external magnetic field of *H*_Z_ = 34 G after ZFC of the sample to 7 K. The blue region exhibits a noticeable diamagnetism. *In situ* Raman measurements are performed on the superconducting regions (B1, B2) and the non-superconducting regions (B3, B5) under different pressures. (c) Magnetic field mapping of one of the superconducting regions [position of purple square marked in (b)] during the FW process; the external magnetic field is around 120 G. Above the critical temperature *T*_c_, the local diamagnetism disappears and all NV centers feel the uniform external magnetic field. The region on the gray square shows a feeble contrast to fit the ODMR splitting. (d) The Raman spectra of B1 and B3 at 20 GPa. As shown in the figure, a small satellite peak is observed in the non-superconducting region (B3) at 20 GPa, while it is suppressed in the superconducting region (B1). This peak is also presented at atmospheric pressure before compression (shown in Fig. S6a).

To investigate the effects of pressure-transmitting media, another La_2_PrNi_2_O_7_ sample (sample B) was loaded in KBr (a solid pressure-transmitting medium) and compressed to 30 GPa. We then characterized the sample with both ODMR and Raman measurements. As shown in Fig. 3a, the confocal image clearly shows the shape and relative position of the sample, the microwave wire, and the edge of the sample chamber. Figure 3b shows a magnetic field image under an external magnetic field of about 34 G after zero-field cooling of the sample to 7 K. The test area of this sample is limited to the area next to the microwave antenna to maintain a good ODMR contrast at low microwave power. Local diamagnetism (reduction of ODMR splitting) was observed in two regions of the sample. The local diamagnetism of one region was tracked during the field-warming (FW) process (under an external magnetic field of around 120 G), as shown in Fig. 3c. As the temperature increases to *T*_c_, the diamagnetic effect gradually fades out. When the temperature is above *T*_c_, the entire region exhibits a uniform magnetic field in line with the strength at reference point B0. More results of the ZFC-FW measurement in sample B can be found in Fig. S4. Compared to sample A, the diamagnetism effect in sample B is smaller. This is plausible because a liquid pressure-transmitting medium (silicon oil) was used in sample A, therefore a better hydrostatic condition was obtained. As a result, a larger superconducting volume and more pronounced diamagnetism were observed in sample A. These results are consistent with the previous studies on the electrical resistance [7,13,14,34].

Thanks to the high spatial resolution of the NV-based magnetometer, the distribution of the superconducting regions is visually obtained, which enables us to perform targeted spectroscopic measurements. We then used the *in situ* Raman spectrum to study the structure of pressurized La_2_PrNi_2_O_7_ in both the superconducting and non-superconducting regions, which are marked in Fig. 3b. As shown in Fig. 3d and Fig. S5b, a slight difference is observed in the Raman spectra, where a satellite peak around 680 cm^−1^ is suppressed in the superconducting region at 20 GPa. The satellite peak is more pronounced in the non-superconducting region and in the sample before compression (see Fig. S6a).

Our density-functional perturbation theory (DFPT) calculations show that structural transformations in La_3_Ni_2_O_7_ manifest as degeneracy of Raman peaks. When La_3_Ni_2_O_7_ is compressed from 0 GPa to about 9 GPa, the difference between the B_2g_ and B_1g_^2^ modes gradually becomes negligible, and a single mode (E_g_) appears under high pressures, as shown in Fig. S6b. The same phenomenon is observed for Pr_3_Ni_2_O_7_ (Fig. S6c), implying that the above discussion may reflect the common feature of this class of materials. Indeed, a similar phenomenon has been observed for the bilayer Ruddlesden–Popper (R–P) perovskite Li_2_CaTa_2_O_7_, which has a similar structure to La_2_PrNi_2_O_7_ [35]. A comparison with the Raman spectra of related compounds [36] shows that the satellite peak in La_2_PrNi_2_O_7_ can be assigned to an oxygen B_1g_ mode. Therefore, the satellite peak in the non-superconducting region may be attributed to the incomplete structural transformation caused by the displacements of the oxygen ions, which is challenging to be observed in X-ray and neutron diffraction [37]. These results are well consistent with the change of the bond angle of the Ni–O–Ni in La_3_Ni_2_O_7_. In the case of La_3_Ni_2_O_7_, the emergence of HTSC is seen along with the structural transformations from *Amam* to *Fmmm* with the change of the bond angle of the Ni–O–Ni from 168.0° to 180° along the c-axis [34]. As a consequence, the electronic interactions within the bilayer of NiO_2_ are increased, corresponding to the metallization of the inter-layer σ-bonding bands. This phenomenon is observed in the conventional high-*T*_c_ superconductors, such as MgB_2_ and Li_3_B_4_C_2_ [7].

### Local diamagnetism imaging in La_3_Ni_2_O_7–δ_

**Figure 4.**
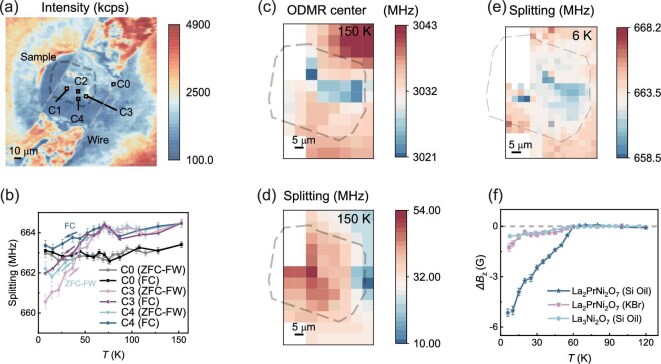
Magnetic and stress measurement of the La_3_Ni_2_O_7–δ_ sample at 20 GPa. (a) Fluorescence image of sample C (La_3_Ni_2_O_7–δ_ in silicon oil). The edge of the sample is outlined by the black dashed line. (b) ODMR splitting of the NV centers during the ZFC-FW and FC processes, with an external magnetic field of around 120 G. The NV positions are marked in (a). Both the diamagnetism of the La_3_Ni_2_O_7–δ_ sample and the local stress contribute to the ODMR splitting. (c, d) Stress distribution revealed by the zero-field ODMR spectra at 150 K (>*T*_c_). The center frequency of the ODMR spectra (c) reveals the compressive stress, while the splitting of the ODMR spectra (d) is proportional to the differential stress. (e) Magnetic image under an external magnetic field of around 120 G after ZFC. Note that the contribution of the stress distribution has been subtracted by the results shown in (c and d). The region on the gray square is an odd point and is therefore not included in the discussion. (f) Comparison of the diamagnetism effect of the three samples during ZFW-FW measurement. The external magnetic field is around 120 G. For each point, the ODMR splitting at high temperatures (>*T*_c_) is used as the reference to calculate *ΔB*_Z_.

To investigate the Meissner effect of La_3_Ni_2_O_7–δ_, a polycrystalline sample was loaded with silicon oil as the pressure-transmitting medium (sample C). Figure 4a shows the confocal image of NV fluorescence when the sample is compressed to 20 GPa. Following the experimental protocols shown in Fig. 2e, the ZFC-FW and FC measurements were performed under an external magnetic field of around 120 G. As shown in Fig. 4b, local diamagnetism was observed during both the FW and FC processes. However, the diamagnetism signal (change of ODMR splitting below and above *T*_c_) in sample C is only one-fifth of sample A, indicating a relatively small superconducting volume in this sample.

Notably, there are obvious differences of ODMR splitting among different NV centers, even when the temperature is above *T*_c._ This phenomenon can be explained by the inhomogeneous distribution of stress on the diamond culet. In particular, the compressive stress can shift the center frequency of the ODMR spectra, while the differential stress regulates the ODMR splitting. It is worth noting that this phenomenon also occurs in the former two samples, but the contribution of stress is much weaker compared to the contribution of the superconducting diamagnetism effect in these two samples. Fortunately, diamond NV centers can also serve as *in situ* stress sensors. To decouple the contribution of stress, we perform ODMR measurements at 150 K (above *T*_c_) in zero field (see Supplementary data for details). As shown in Fig. 4c and d, the maximum pressure variance among the sample is about 3 GPa. With this in hand, we can eliminate the influence of non-uniform stress distribution and obtain a strain-free magnetism distribution, as shown in Fig. 4e. In short, the Meissner effect of La_3_Ni_2_O_7–δ_ was imaged with NV centers under high pressure.

Finally, the diamagnetism signals of three samples during ZFC-FW are normalized and compared with each other. As shown in Fig. 4f, it can be clearly seen that pressurized La_2_PrNi_2_O_7_ in silicon oil exhibits the strongest diamagnetism effect, while La_2_PrNi_2_O_7_ in KBr has a relatively weaker signal, indicating that the hydrostatic conditions play an important role in achieving a high superconducting volume fraction. Overall, La_3_Ni_2_O_7–δ_ in silicon oil has the weakest signal, which is consistent with its inhomogeneous nature (mixture of La_3_Ni_2_O_7_ and other phases such as La_4_Ni_3_O_10_ and La_2_NiO_4_) [24,25].

## DISCUSSION

It is noteworthy that the superconducting diamagnetism observed in our work exhibits a weaker magnitude compared to other superconducting systems (e.g. cuprate and hydride superconductors). We attribute this weaker diamagnetic response primarily to the limited superconducting volume fraction in the measured nickelate samples. The same situation has occurred in previous electrical and magnetic measurements of nickelate, which have consistently reported relatively weak superconducting signals with pronounced sample inhomogeneity [7,11,13,14,26]. Although there is no perfect diamagnetic signal, the characteristic field-dependent magnetic response and the expulsion of magnetic flux during FC measurements clearly confirm the presence of the Meissner effect in pressurized bilayer nickelate.

In conclusion, the Meissner effect is imaged in pressurized bilayer nickelate superconductors La_3_Ni_2_O_7–δ_ and La_2_PrNi_2_O_7_ using NV center quantum sensors. By combining ZFC-FW measurements with FC measurements in different external magnetic fields, our results confirm the existence of HTSC in La_3_Ni_2_O_7–δ_ and La_2_PrNi_2_O_7_. Through the correlated measurement between ODMR and Raman spectroscopy, we identify the structural difference between superconducting and non-superconducting regions in the La_2_PrNi_2_O_7_ sample. These measurements indicate that the inhomogeneous superconductivity may be due to the incomplete structural transformations related to the displacement of oxygen ions on the non-superconducting regions and the complete transformations on the superconducting regions, which helps us to understand the underlying mechanism in nickelate high-temperature superconductors.

Our research demonstrates that the diamond NV center is a powerful probe to study inhomogeneous samples under high pressure, due to their high sensitivity, high spatial resolution and multi-parameter sensing capacity. When measuring samples with weak magnetic signals under high pressure, the influence of the stress gradient is a critical issue that should be given sufficient attention. With spatially resolved ODMR measurement, various *in situ* high-pressure experiments, such as Raman and absorption spectroscopy, can be performed efficiently. Our work not only demonstrates the feasibility of using NV center sensors to measure weak magnetic signals under high pressure but also provides a detailed experimental methodology.

## Supplementary Material

nwaf268_Supplemental_File

## References

[1] Li D, Lee K, Wang BY et al. Superconductivity in an infinite-layer nickelate. Nature 2019; 572: 624–7. 10.1038/s41586-019-1496-5 31462797

[2] Zeng S, Tang CS, Yin X et al. Phase diagram and superconducting dome of infinite-layer Nd_1−x_Sr_x_NiO_2_ thin films. Phys Rev Lett 2020; 125: 147003. 10.1103/PhysRevLett.125.147003 33064530

[3] Zeng S, Li C, Chow LE et al. Superconductivity in infinite-layer nickelate La_1−x_Ca_x_NiO_2_ thin films. Sci Adv 2022;8: eabl9927. 10.1126/sciadv.abl9927 35179968 PMC8856608

[4] Ding X, Tam CC, Sui X et al. Critical role of hydrogen for superconductivity in nickelates. Nature 2023; 615: 50–5. 10.1038/s41586-022-05657-2 36859583

[5] Osada M, Wang BY, Lee K et al. Phase diagram of infinite layer praseodymium nickelate Pr_1−x_Sr_x_NiO_2_ thin films. Phys Rev Mater 2020;4: 121801. 10.1103/PhysRevMaterials.4.121801

[6] Wang NN, Yang MW, Yang Z et al. Pressure-induced monotonic enhancement of *T*_c_ to over 30 K in superconducting Pr_0.82_Sr_0.18_NiO_2_ thin films. Nat Commun 2022; 13: 4367. 10.1038/s41467-022-32065-x 35902566 PMC9334608

[7] Sun H, Huo M, Hu X et al. Signatures of superconductivity near 80 K in a nickelate under high pressure. Nature 2023; 621: 493–8. 10.1038/s41586-023-06408-7 37437603

[8] Christiansson V, Petocchi F, Werner P et al. Correlated electronic structure of La_3_Ni_2_O_7_ under pressure. Phys Rev Lett 2023; 131: 206501. 10.1103/PhysRevLett.131.206501 38039471

[9] Zhang Y, Lin L-F, Moreo A et al. Structural phase transition, *s*_±_-wave pairing, and magnetic stripe order in bilayered superconductor La_3_Ni_2_O_7_ under pressure. Nat Commun 2024; 15: 2470. 10.1038/s41467-024-46622-z 38503754 PMC10951331

[10] Liu Z, Huo M, Li J et al. Electronic correlations and partial gap in the bilayer nickelate La_3_Ni_2_O_7_. Nat Commun 2024; 15: 7570. 10.1038/s41467-024-52001-5 39217168 PMC11365989

[11] Zhu Y, Peng D, Zhang E et al. Superconductivity in pressurized trilayer La_4_Ni_3_O_10-δ_ single crystals. Nature 2024; 631: 531–6. 10.1038/s41586-024-07553-3 39020034

[12] Wang L, Li Y, Xie S-Y et al. Structure responsible for the superconducting state in La_3_Ni_2_O_7_ at high-pressure and low-temperature conditions. J Am Chem Soc 2024; 146: 7506–14. 10.1021/jacs.3c13094 38457476

[13] Hou J, Yang P-T, Liu Z-Y et al. Emergence of high-temperature superconducting phase in pressurized La_3_Ni_2_O_7_ crystals. Chin Phys Lett 2023; 40: 117302. 10.1088/0256-307X/40/11/117302

[14] Zhang Y, Su D, Huang Y et al. High-temperature superconductivity with zero resistance and strange-metal behaviour in La_3_Ni_2_O_7−δ_. Nat Phys 2024; 20: 1269–73. 10.1038/s41567-024-02515-y

[15] Wang G, Wang NN, Shen XL et al. Pressure-induced superconductivity in polycrystalline La_3_Ni_2_O_7−δ_. Phys Rev X 2024; 14: 011040.

[16] Yang J, Sun H, Hu X et al. Orbital-dependent electron correlation in double-layer nickelate La_3_Ni_2_O_7_. Nat Commun 2024; 15: 4373. 10.1038/s41467-024-48701-7 38782908 PMC11116484

[17] Abadi S, Xu K-J, Lomeli EG et al. Electronic structure of the alternating monolayer-trilayer phase of La_3_Ni_2_O_7_. Phys Rev Lett 2025; 134: 126001. 10.1103/PhysRevLett.134.126001 40215506

[18] Xie T, Huo M, Ni X et al. Strong interlayer magnetic exchange coupling in La_3_Ni_2_O_7–δ_ revealed by inelastic neutron scattering. Sci Bull 2024; 69: 3221–7. 10.1016/j.scib.2024.07.030 39174404

[19] Khasanov R, Hicken TJ, Gawryluk DJ et al. Pressure-enhanced splitting of density wave transitions in La_3_Ni_2_O_7−δ_. Nat Phys 2025; 21: 430–6. 10.1038/s41567-024-02754-z

[20] Chen K, Liu X, Jiao J et al. Evidence of spin density waves in La_3_Ni_2_O_7–δ_. Phys Rev Lett 2024; 132: 256503. 10.1103/PhysRevLett.132.256503 38996236

[21] Zhao D, Zhou Y, Huo M et al. Pressure-enhanced spin-density-wave transition in double-layer nickelate La_3_Ni_2_O_7–δ_. Sci Bull 2025; 70: 1239–45. 10.1016/j.scib.2025.02.019 40016038

[22] Puphal P, Reiss P, Enderlein N et al. Unconventional crystal structure of the high-pressure superconductor La_3_Ni_2_O_7_. Phys Rev Lett 2024; 133: 146002. 10.1103/PhysRevLett.133.146002 39423411

[23] Zhou Y, Guo J, Cai S et al. Investigations of key issues on the reproducibility of high-*T*_c_ superconductivity emerging from compressed La_3_Ni_2_O_7_. Matter Radiat Extremes 2025; 10: 027801. 10.1063/5.0247684

[24] Li J, Peng D, Ma P et al. Identification of the superconductivity in bilayer nickelate La_3_Ni_2_O_7_ upon 100 GPa. Natl Sci Rev 2025; 12: nwaf220. 10.1093/nsr/nwaf220 PMC12485610 41040486

[25] Dong Z, Huo M, Li J et al. Visualization of oxygen vacancies and self-doped ligand holes in La_3_Ni_2_O_7−δ_. Nature 2024; 630: 847–52. 10.1038/s41586-024-07482-1 38839959

[26] Wang N, Wang G, Shen X et al. Bulk high-temperature superconductivity in pressurized tetragonal La_2_PrNi_2_O_7_. Nature 2024; 634: 579–84. 10.1038/s41586-024-07996-8 39358510

[27] Hsieh S, Bhattacharyya P, Zu C et al. Imaging stress and magnetism at high pressures using a nanoscale quantum sensor. Science 2019; 366: 1349–54. 10.1126/science.aaw4352 31831662

[28] Lesik M, Plisson T, Toraille L et al. Magnetic measurements on micrometer-sized samples under high pressure using designed NV centers. Science 2019; 366: 1359–62. 10.1126/science.aaw4329 31831664

[29] Shang Y-X, Hong F, Dai J-H et al. Magnetic sensing inside a diamond anvil cell via nitrogen-vacancy center spins. Chin Phys Lett 2019; 36: 086201. 10.1088/0256-307X/36/8/086201

[30] Yip KY, Ho KO, Yu KY et al. Measuring magnetic field texture in correlated electron systems under extreme conditions. Science 2019; 366: 1355–59. 10.1126/science.aaw4278 31831663

[31] Bhattacharyya P, Chen W, Huang X et al. Imaging the Meissner effect in hydride superconductors using quantum sensors. Nature 2024; 627: 73–9. 10.1038/s41586-024-07026-7 38418887

[32] Wang M, Wang Y, Liu Z et al. Imaging magnetism evolution of magnetite to megabar pressure range with quantum sensors in diamond anvil cell. Nat Commun 2024; 15: 1. 10.1038/s41467-024-52768-7 39397023 PMC11471789

[33] Dai J-H, Shang Y-X, Yu Y-H et al. Optically detected magnetic resonance of diamond nitrogen-vacancy centers under megabar pressures. Chin Phys Lett 2022; 39: 117601. 10.1088/0256-307X/39/11/117601

[34] Wang M, Wen H-H, Wu T et al. Normal and superconducting properties of La_3_Ni_2_O_7_. Chin Phys Lett 2024; 41: 077402. 10.1088/0256-307X/41/7/077402

[35] Galven C, Mounier D, Bouchevreau B et al. Phase transitions in the Ruddlesden–Popper Phase Li_2_CaTa_2_O_7_: X-ray and neutron powder thermodiffraction, TEM, Raman, and SHG experiments. Inorg Chem 2016; 55: 2309–23. 10.1021/acs.inorgchem.5b02659 26901319

[36] Dias A, Viegas JI, Moreira, RL. Synthesis and μ-Raman scattering of Ruddlesden-Popper ceramics Sr_3_Ti_2_O_7_, SrLa_2_Al_2_O_7_ and Sr_2_LaAlTiO_7_. J Alloys Compd 2017; 725: 77–83. 10.1016/j.jallcom.2017.07.155

[37] Adler P, Goncharov AF, Syassen K et al. Optical reflectivity and Raman spectra of Sr_2_FeO_4_ under pressure. Phys Rev B 1994; 50: 11396–402. 10.1103/PhysRevB.50.11396 9975270

