# Supplementary material for: Imaging the Meissner effect in pressurized bilayer nickelate with integrated multi-parameter quantum sensor
Source: Natl Sci Rev. 2025 Jul 4;12(10):nwaf268. doi: 10.1093/nsr/nwaf268 (PMC12485607; doi:10.1093/nsr/nwaf268)
Supplement: nwaf268_Supplemental_File [file nwaf268_supplemental_file.pdf]

Supplementary Information for

Imaging the Meissner effect in pressurized bilayer nickelate with integrated multi-parameter quantum sensor

Junyan Wen[1,2†], Yue Xu[1,2†], Gang Wang[1,2†], Ze-Xu He[1,2], Yang Chen[1], Ningning Wang[1,2], Tenglong Lu[1], Xiaoli Ma[1,2], Feng Jin[1,2], Liucheng Chen[1], Miao Liu[1], Jing-Wei Fan[3], Xiaobing Liu[4], Xin-Yu Pan[1,2,5], Gang-Qin Liu[1,2,5*], Jinguang Cheng[1,2*], Xiaohui Yu[1,2*]

[1]*Beijing National Laboratory for Condensed Matter Physics and Institute of Physics, Chinese Academy of Sciences, Beijing 100190, China*

[2]*School of Physical Sciences, University of Chinese Academy of Sciences, Beijing 100190, China*

[3] *Department of Physics, Hefei University of Technology, Hefei, Anhui 230009, China*

[4] *Laboratory of High Pressure Physics and Material Science, School of Physics and Physical Engineering, Qufu Normal University, Qufu, Shandong 273165, China*

[5] *CAS Center of Excellence in Topological Quantum Computation, Beijing 100190, China*

† These authors contribute equally to this work.

*Corresponding authors: gqliu@iphy.ac.cn; jgcheng@iphy.ac.cn; yuxh@iphy.ac.cn

Supplementary figures:

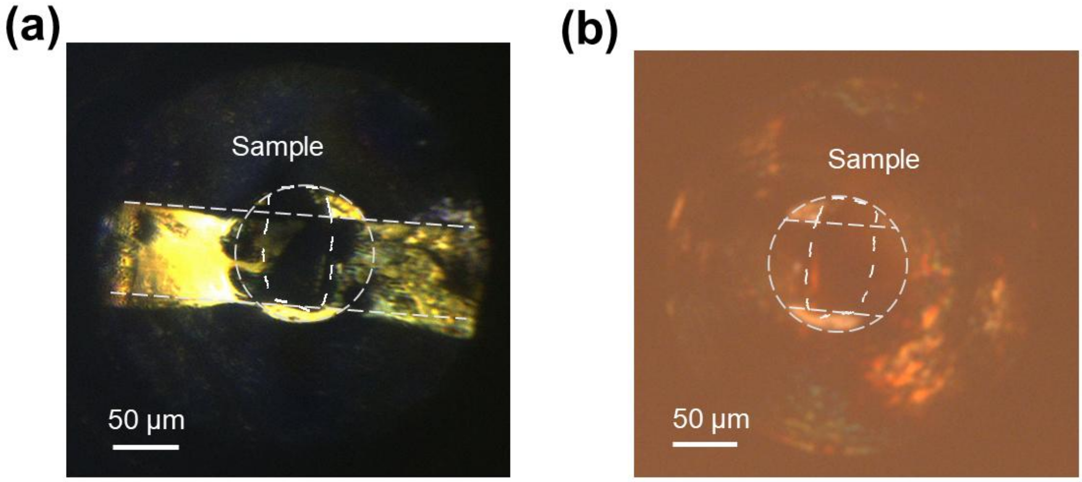

Fig. S1 Bright field image of sample A from the top view (a) and the bottom view (b). The sample, chamber and the wire are marked in the figure. The profile of the sample is determined from the brightfield image of the bottom view.

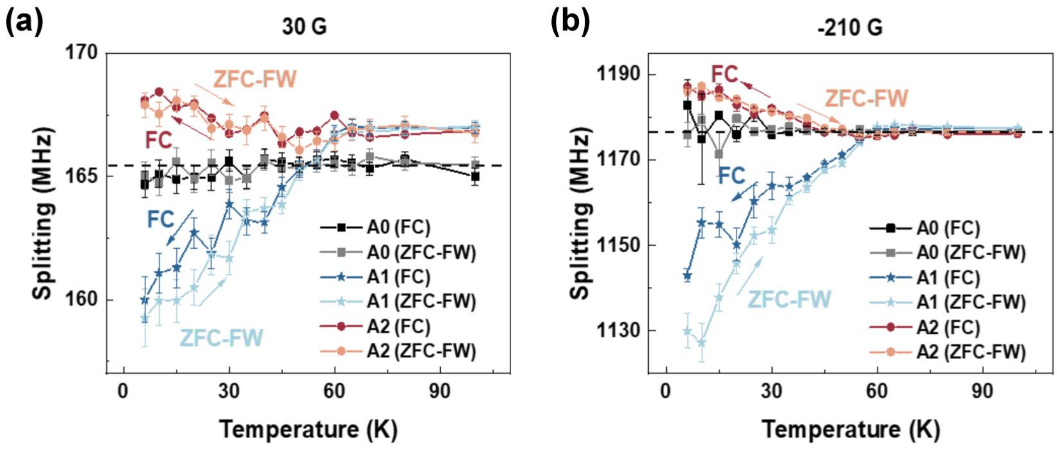

Fig. S2 Diamagnetism of sample A ($La_2PrNi_2O_7$ in silicon oil) under different external magnetic fields. ODMR splitting during the ZFC-FW and FC processes of sample A at 20 GPa and an external magnetic field of 30 G **(a)** and -210 G **(b).** The measured points (A0, A1 and A2) are marked in Fig. 2a of the main text. The measurement protocol is shown in Fig. 2e of the main text.

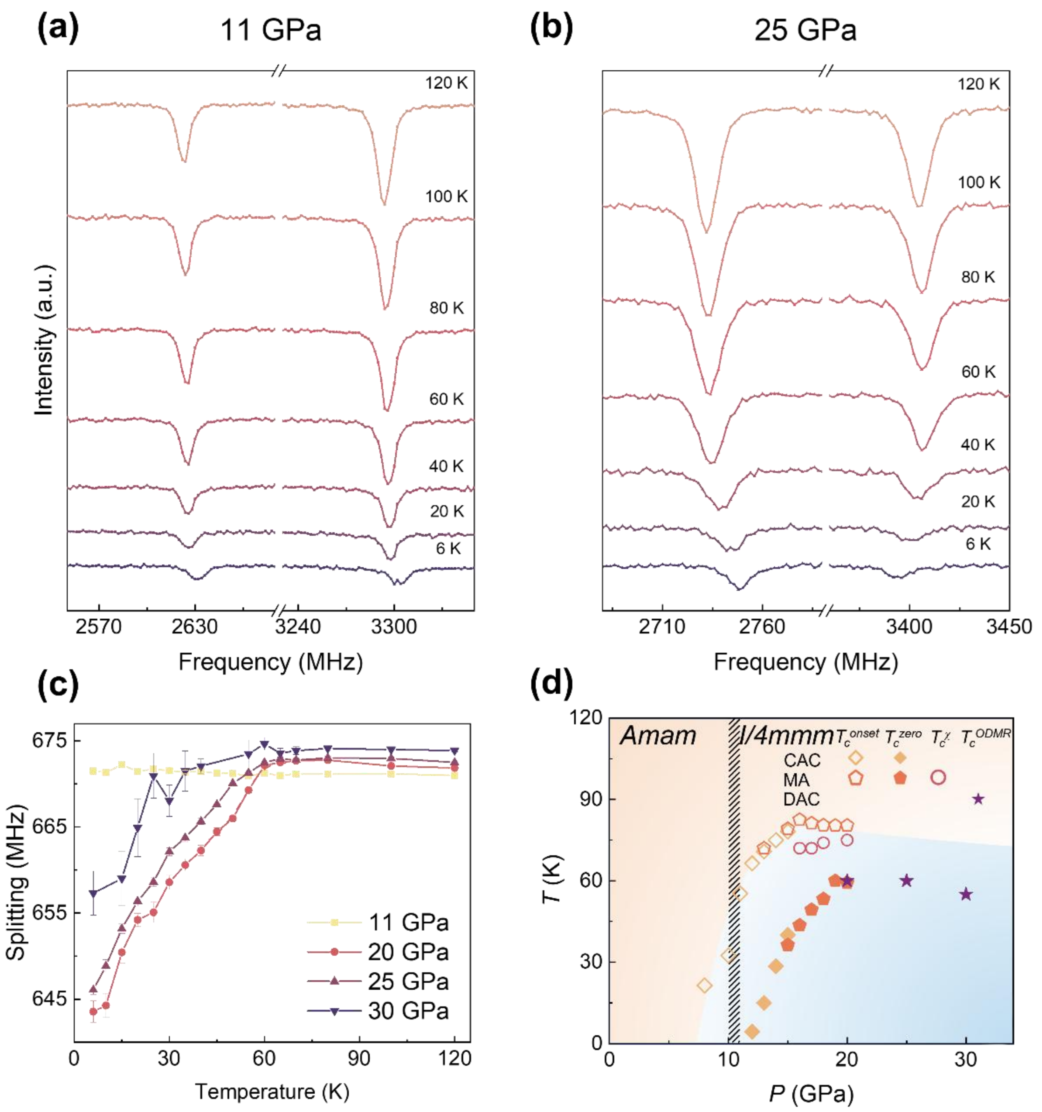

Fig. S3 ODMR spectra of sample A ($La_2PrNi_2O_7$ in silicon oil) at different pressures. The ODMR spectra are measured at point A1 of sample A during the ZFC-FW process and at an external magnetic field of 120 G. The pressure is 11 GPa and 25 GPa for **(a)** and **(b)**, respectively. **(c)** ODMR splitting of point A1 at different pressures during the ZFC-FW measurement at 120 G. At a pressure of 11 GPa, sample A shows no diamagnetic effect. The sample shows maximum diamagnetism at 20 GPa and gradually decreases with increasing pressure. **(d)** The *T-P* phase diagram of $La_2PrNi_2O_7$. The purple star represents the T_c value determined from the ODMR measurement. The rhomboid, pentagon and circle are the T_c values obtained in reference [1,2].

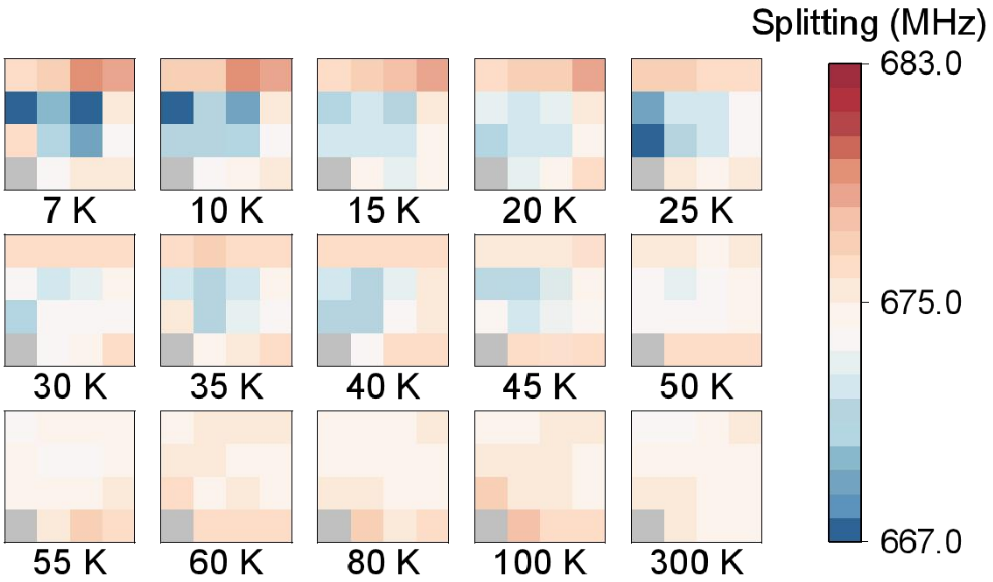

Fig. S4 Magnetic field image of sample B ($La_2PrNi_2O_7$ in KBr) during the ZFC-FW process. Magnetic field image of a superconducting region on sample B ($La_2PrNi_2O_7$ in KBr) during the FW process. The exact position of the measured region is marked in Fig. 3b of the main text. The external magnetic field is 120 G. Part of the results are shown in Fig. 3c of the main text. The point shown in the gray square is not considered in the discussion because its ODMR contrast is too low.

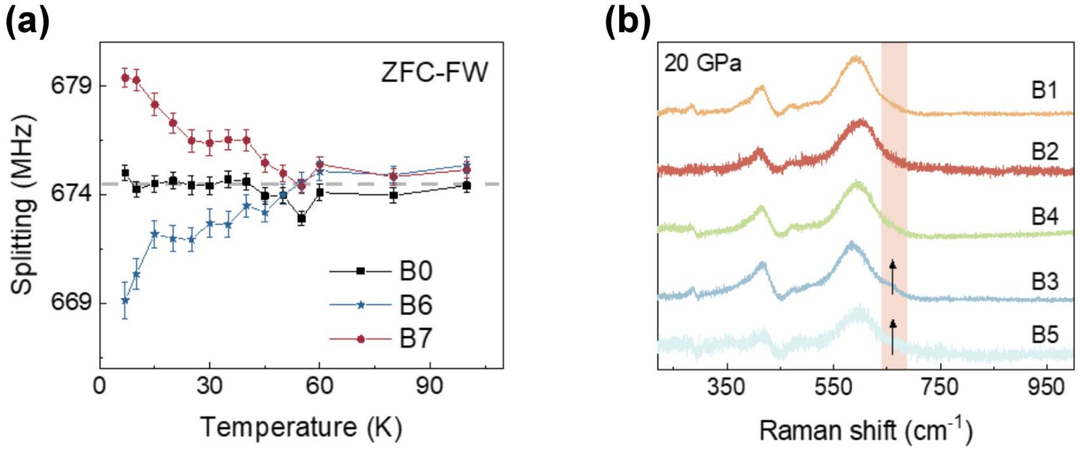

Fig. S5 Diamagnetism and Raman spectra of sample B ($La_2PrNi_2O_7$ in KBr) (a) ODMR measurement at 30 GPa during the ZFC-FW process. **(b)** Raman spectra measured at the test points of sample B at 20 GPa. The position of the measured points is shown in Fig. 3b of the main text. Points B1, B2 and B4 are located in the superconducting region, B3 and B5 in the non-superconducting region. A satellite peak around 680 cm^{-1} is observed in the Raman spectra of B3 and B5, while it is suppressed in the Raman spectra of B1, B2 and B4.

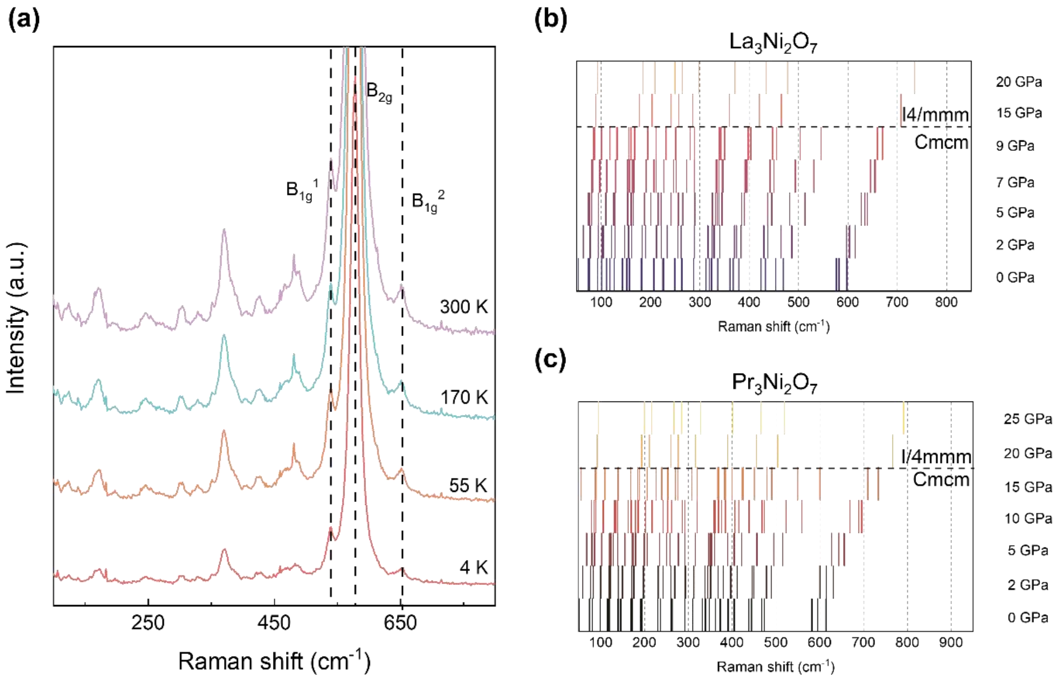

Fig. S6 Raman spectrum of $La_2PrNi_2O_7$ at ambient pressure and the DFPT calculations results. **(a)** Raman spectrum measured with a 785-nm laser on the $La_2PrNi_2O_7$ sample at ambient pressure and different temperatures. The Raman spectrum is offset in the Y-axis for clarity. **(b-c)** DFPT calculations results: Raman peaks at 0 K of $La_3Ni_2O_7$ **(b)** and $Pr_3Ni_2O_7$ **(c)** at different pressures. The three highest Raman-active vibrational modes B_{1g}^1, B_{2g}, and B_{1g}^2 are shown accordingly in **(a)**. With increasing pressure, these three modes gradually shift to higher frequencies and the splitting between the B_{2g} and B_{1g}^2 modes becomes negligible. In the high-pressure structure, there is only one Raman-active mode (E_g) due to the degeneracy of the B_{2g} and B_{1g}^2 modes.

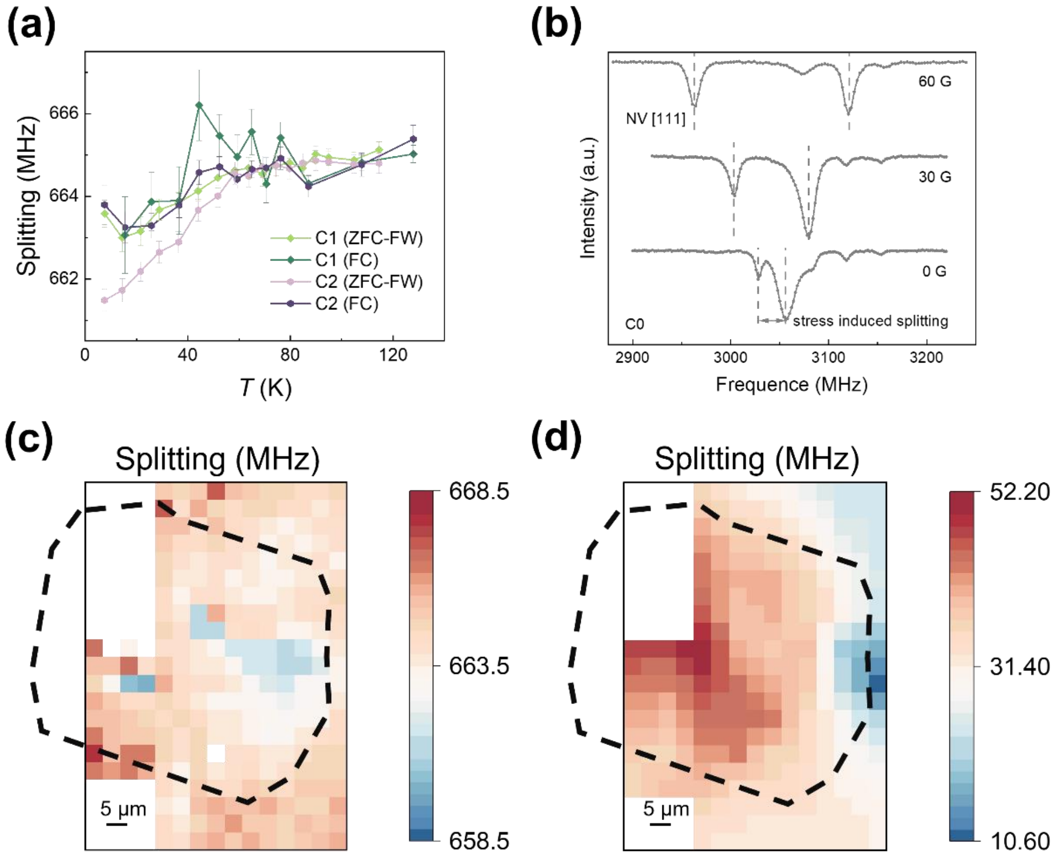

Fig. S7 ODMR splitting and stress distribution of sample C ($La_3Ni_2O_{7-\delta}$ in silicon oil). (a) ODMR splitting of two points (C1 and C2) during the ZFC-FW and FC processes with an external magnetic field of 120 G. The positions of the points are shown in Fig. 4a of the main text. (b) ODMR spectra of reference point C0 at different magnetic fields. The ODMR dips marked with the dashed line correspond to the NV centers along the [111] orientation, which shift linearly with the external magnetic field due to the Zeeman effect. At zero field, the ODMR splitting due to transvers stress can be estimated. **(c)** Spatial distribution of ODMR splitting at 120 G and 6 K. Both the diamagnetism and the local stress of sample C contribute to the ODMR splitting. **(d)** Spatial distribution of ODMR splitting at 150 K and 0 G. By combining **(c)** and **(d)**, we can decouple the contribution of stress and magnetic field, and the purely magnetic field distribution around sample C is shown in Fig. 4e of the main text.

Methods:

1. Sample synthesis.

Polycrystalline $La_2PrNi_2O_7$ and $La_3Ni_2O_{7-\delta}$ samples were synthesized using the sol-gel method with identical procedures as described in reference [1,2]. Stoichiometric mixtures of rare-earth oxides and $Ni(NO_3)_2\cdot6H_2O$ with a purity of 99.99% from Alfa Aesar were dissolved in deionized water together with citric acid and nitric acid. The solution was stirred in a 90 °C water bath for approximately 4 hours, resulting in a vibrant green nitrate gel. The gel was then heat-treated overnight at 800 °C to remove excess organic matter. The product was then ground, pressed into pellets, and sintered in air at 1100-1150 °C for 24 hours.

2. Sample preparation.

The pressure was generated using a diamond anvil cell (DAC) made of a non-magnetic BeCu alloy. All experiments were carried out on standard diamond anvils with [111]-crystal cut. These anvils were polished from synthetic type-IIa single crystal diamonds. The diameter of the diamond anvil culet was 300 μm. N ions with an energy of 20 keV and dosage of 2×10^{14} cm^{-2} were implanted on the diamond culet to create a layer of vacancies. After implantation, diamond anvils were annealed at 800 °C for 2 hours to form NV centers. A rhenium gasket with an initial thickness of 200 μm was pre-intended to 40 μm, then a 120 μm-diameter hole was drilled in the center with a laser beam. After ultrasonic cleaning, the gasket was coated with a layer of insulating glue while the hole was covered with a layer of aluminium oxide (Al_2O_3) powder. The aluminium oxide powder was tightly compacted and further drilled to form the high-pressure chamber (with a diameter of about 100 μm). Polycrystalline $La_2PrNi_2O_7$ and $La_3Ni_2O_{7-\delta}$ samples were cut into pieces of 100 μm × 60 μm × 40 μm and 40 μm × 50 μm × 30 μm, respectively, and placed into the chamber. Silicon oil (samples A and C) or KBr disk (sample B) was then loaded into the chamber and served as the pressure transmitting media. A 0.5-μm thick gold (Au) foil was cut in strips and placed on the diamond to transmit microwave pulses. The Raman spectra of the diamond and the zero-field splitting of NV were used to calibrate the pressure.

3. ODMR measurement.

Optically detected magnetic resonance (ODMR) measurements were performed with a custom-built confocal microscopy system. A 532-nm laser (Changchun New Industries) was used

to polarize and read out the spin states of shallow NV centers in the surface layer of the DAC culet. The laser beam was focused on these NV centers through an objective lens with long working distance. The spin-dependent fluorescence emitted by the NV centers was collected by the same objective lens, filtered in the 650-800 nm range, and detected by a single-photon counting module (Excelitas, SPCM-NIR). The counting signal was then recorded and sent to a computer via a data acquisition card (National Instruments, USB-6343). The focus position of the confocal microscopy was controlled by a pair of scanning mirrors, while the focus depth was controlled by a piezo-stage. The laser pulses were generated using an acousto-optic modulator (G&H, AOM), and microwave pulses were controlled by an RF switch (Mini-circuits, ZASW-2-50DRA+). The SPCM, AOM and RF switch were controlled by a programmable multi-channel pulse generator (SpinCore PulseBlasterESR-PRO 500) with temporal resolution down to 2 ns. Some components, including the DAC, objective lens and piezo stage, were housed in a cryostat (Montana Instruments, s100). The samples temperature can be cooled down to 6 K, with a fluctuation of less than 50 mK. The ODMR spectra were recorded in continuous-wave mode, wherein laser excitation, microwave (MW) driving, and photon counting simultaneously performed while the frequency of MW pulses was scanned. Each MW frequency was counted for a duration of 1 ms, evenly divided between signal counts and reference counts when the MW control was switched on or off. This sequence was repeated until a reasonable signal-to-noise ratio was achieved, and the counts of each MW frequency were summed up and normalized by their respective reference counts. In this way, we can effectively eliminate slow fluctuations caused by laser power instability (< 1%) and sample position drift.

4. Raman measurement.

In-situ high pressure Raman spectrum was performed with a backscattering configuration using a Jobin Yvon HR-Evolution system consisting of a diode-pumped solid state 785-nm laser. The excitation laser beam was focused into a spot of ~ 5 μm in diameter. The low-temperature high-pressure Raman spectra were collected using an S&I MonoVista CRS+ 750 System equipped with a liquid-nitrogen-cooled charge-coupled device (CCD). A 785 nm laser was used, with a spot size of 1 ~ 2 μm. The laser power was maintained at a low power (~ 3 mW) to avoid overheating during the measurements.

5. Theory (DFT) calculations.

Density functional theory (DFT) calculations were conducted using the projector-augmented wave (PAW) method, implemented in the Vienna *ab initio* simulation package (VASP) [3,4]. All calculations utilized the Perdew-Burke-Ernzerhof (PBE) generalized gradient approximation (GGA) exchange-correlation functional [5]. To correct for the self-interaction error on the Ni species, a rotationally averaged Hubbard U correction of 3 eV was applied [6]. For all calculations, a plane wave energy cutoff of 520 eV, an electronic minimization threshold of 10^{-6} eV, and a k-point grid of $n_{kpoints} \times n_{atoms} > 1000$ were adopted. All DFT calculations in this study were performed without spin-polarization. The vibrational frequencies and mode eigenvectors at the zone center Γ point was computed using the density-functional perturbation theory (DFPT).

Supplementary notes:

1. Assignment of the NV centers' orientation.

The NV centers with the *right* orientation (which match the (111) cutting orientation) are picked up by tuning the orientation and strength of the external magnetic field. A permanent magnet outside the cryostat chamber is used to generate the magnetic field. The strength and orientation of the magnetic field are controlled by a translation stage and two rotation stages. The experiments are performed in such a way that the magnetic field is perpendicular to the DAC culet, i.e. parallel to the quantization axis of a group of the shallow NV centers. The ODMR spectra are measured at different magnetic field strengths, as shown in Fig. S7b. As the magnetic field increases, the NV centers with [111] orientation split symmetrically due to the Zeeman effect, and the center frequencies of the two dips remain unchanged. In contrast, for NV centers in other orientations, their ODMR splitting is largely suppressed as they experience a strong transverse stress. The strength of the external magnetic field is calibrated with NV centers that are far away from the sample region.

2. Calibration of pressures.

We use two methods to measure the pressures at the sample position. First, the Raman signals of the diamond culet above the sample are measured with a 532 nm laser to calibrate the pressure. The high-frequency edge of the Raman band, which corresponds to the Raman shift of the anvil

culet due to the normal stress, is calculated by the pressure equation of state reformulated from the pressure-density relation of diamond [7].

$$P(\mathrm{GPa}) \cong P_0 \frac{\Delta \nu}{\nu_0}\left[1+\frac{1}{2}(K-1)\frac{\Delta \nu}{\nu_0}\right] \tag{S1}$$

where Δν and ν_0 correspond to the detected diamond frequency shift and the frequency at ambient pressure. P_0 and K are fitting parameters related to the pressure environment. The values of P_0 = 547 GPa and K = 3.75 [8]. Using the analytical form of equation (S1) with the edge frequency ν_0 = 1330 cm^{-1} measured at ambient pressure, we can obtain the pressure on the sample.

Another method is the use of NV centers as in-situ pressure sensors. After pressurizing or depressurizing the sample, the ODMR spectra of NV centers at the target position are measured. The resonance dips of the ODMR spectra, f_{+1} and f_{-1}, are extracted, and the center frequency $f_c = (f_{+1} + f_{-1})/2$ is used to calculate the pressure P according to the following formula.

$$P = \frac{f_c - f_{c0}}{\alpha} \tag{S2}$$

where $f_{c0} = 2870\ \mathrm{MHz}$ is the zero-field splitting of NV centers at ambient temperature and pressure, and the slope $\alpha = 7.24\ \mathrm{MHz/GPa}$ [9]. The pressure at point A0 is summarized in Table S1.

Table S1 ODMR resonance dips and pressures at A0 of sample A

Experiment	f_{-1}/MHz	f_{+1}/MHz	f_c/MHz	P/MHz
1	3018.7	3030.3	3024.5	21.3
2	3048.6	3061.2	3054.9	25.5
3	2944.1	2954.9	2949.5	11.0
4	3083.1	3095.5	3089.3	30.3

3. Decoupling of magnetic field and local stress.

The Hamiltonian of NV center ground state can be expressed [10]:

$$H_i/h = (D + M_{Z_i})S_{Z_i}^2 + \gamma_B \vec{B} \bullet \vec{S_i} - M_{X_i}(S_{X_i}^2 - S_{Y_i}^2) + M_{Y_i}(S_{X_i}S_{Y_i} + S_{Y_i}S_{X_i}) \tag{S3}$$

where h is the Plank constant, D = 2.87 GHz is the zero-field splitting, γ_B is the gyromagnetic ratio of NV electron spin, $\vec{S_i} = (S_{X_i}, S_{Y_i}, S_{Z_i})$ are the spin-1 operators, $\vec{B}$ is the magnetic field, and $(M_{X_i}, M_{Y_i}, M_{Z_i})$ are the components of an effective field $\vec{M}$. $\vec{M}$ is a reduction of

stress tensor $\overleftrightarrow{\sigma}$ whose components are obtained as symmetry adapted linear combinations of the stress tensor components. The i = 1, 2, 3, 4 refer to the four possible orientations of NV center. For NV centers with different orientations, their stress components, $\sigma_{\parallel}$ (parallel component) and $\sigma_{\perp}$(perpendicular component), differ from each other. The stress tensor can be simplified as:

$$\overleftrightarrow{\sigma} = \begin{bmatrix} \alpha P & 0 & 0 \\ 0 & \alpha P & 0 \\ 0 & 0 & P \end{bmatrix} \tag{S4}$$

where P is the stress normal to the diamond anvil surface, and α is a parameter quantifying the non-hydrostaticity in the radial directions. Using this simplification of the stress tensor and assuming that the magnetic field is along the [111] direction, then diagonalization of the Hamiltonian in (S3) yields the approximate eigenfrequencies for the NV ground state:

$$f_{\pm 1} = D + M_Z \pm \frac{\Delta}{2} \tag{S5}$$

Magnetic fields and stress contribute simultaneously to the splitting of the ODMR spectra. The total splitting of the ODMR spectra Δ can be expressed as follows: $\Delta = \sqrt{\Delta_{magnetic}^2 + \Delta_{stress}^2} = \sqrt{(2\gamma_B B)^2 + 4M_X^2 + 4M_Y^2}$, where $\Delta_{magnetic}$ represents the splitting caused by the magnetic field, and Δ_{stress} refers to the splitting caused by the off-axis stress (stress that is not aligned along the NV axis). In our experiments, the ODMR spectra of NV centers were measured at zero field and 150 K (above T_C) to determine the contribution of local pressure. The distribution of stress-induced splitting in sample C ($La_3Ni_2O_{7-\delta}$ in silicon oil) is shown in Fig. 4d of the main text. It is clear that the stress-induced ODMR splitting changes significantly in the whole sample region, which is superimposed on the weak demagnetization signal of the $La_3Ni_2O_{7-\delta}$ sample. To solve this problem, we subtract (in quadrature) the stressed component and obtain the pure magnetic splitting shown in Fig. 4e of the main text. Note that a linear interpolation is performed to obtain the non-magnetic splitting with higher spatial resolution to compensate for the different number of data points in Fig. S7c and S7d.

References

1 Wang N, Wang G, Shen X *et al.* Bulk high-temperature superconductivity in pressurized tetragonal $La_2PrNi_2O_7$. *Nature* 2024;**634**:579-584.

2 Wang G, Wang NN, Shen XL *et al.* Pressure-Induced Superconductivity In Polycrystalline $La_3Ni_2O_{7-\delta}$. *Phys Rev X* 2024;**14**:011040.

3 Kresse G and Furthmuller J Efficiency of ab-initio total energy calculations for metals and semiconductors using a plane-wave basis set. *Comp Mater Sci* 1996;**6**:15-50.

4 Kresse G and Joubert D From ultrasoft pseudopotentials to the projector augmented-wave method. *Phys. Rev. B* 1999;**59**:1758-1775.

5 Perdew J P, Burke K and Ernzerhof M Generalized gradient approximation made simple. *Phys. Rev. Lett.* 1996;**77**:3865-3868.

6 Dudarev S L, Botton G A and Savrasov S Y *et al.* Electron-energy-loss spectra and the structural stability of nickel oxide: An LSDA+U study. *Phys. Rev. B* 1998;**57**:1505-1509.

7 Eremets M I Megabar high-pressure cells for Raman measurements. *J. Raman Spectrosc.* 2003;**34**:515-518.

8 Akahama Y and Kawamura H Pressure calibration of diamond anvil Raman gauge to 310 GPa. *J. Appl. Phys.* 2006;**100**:043516.

9 Wang M, Wang Y, Liu Z *et al.* Imaging magnetism evolution of magnetite to megabar pressure range with quantum sensors in diamond anvil cell. *Nat. Commun.* 2004;**15**:1-8.

10 Antoine H, Loïc T, Cassandra D *et al*. Enabling quantum sensing under extreme pressure: Nitrogen-vacancy magnetometry up to 130 GPa. *Phys. Rev. B.* 2023;**107:**L220102.
